# Diffuse sclerosing variant of papillary thyroid carcinoma: outcomes of 33 cases

**DOI:** 10.1530/ETJ-21-0020

**Published:** 2021-12-09

**Authors:** Daniela Cavaco, Ana Filipa Martins, Rafael Cabrera, Helena Vilar, Valeriano Leite

**Affiliations:** 1 Department of Endocrinology, Instituto Português de Oncologia de Lisboa Francisco Gentil, EPE, Lisbon, Portugal; 2 Department of Endocrinology and Diabetes, Hospital da Luz, Lisbon, Portugal; 3 Department of Patology, Instituto Português de Oncologia de Lisboa Francisco Gentil, EPE, Lisbon, Portugal; 4 Nova Medical School, Lisbon, Portugal

**Keywords:** diffuse sclerosing variant, papillary thyroid carcinoma, aggressive thyroid

## Abstract

**Introduction:**

Diffuse sclerosing variant of PTC (DSV-PTC) is an uncommon subtype of thyroid cancer. Although an aggressive behavior is often recognized, prognostic significance is still under debate.

**Objectives:**

To describe the clinicopathological features and outcomes of a series of DSV-PTC patients.

**Methods:**

Retrospective data collection involving 33 patients diagnosed with DSV-PTC followed at the Endocrine Department of the Portuguese Institute of Oncology in Lisbon between 1981 and 2020.

**Results:**

Twenty-six patients (78.8%) were females with a mean age at presentation of 29.4 ± 11.7 years old. Mean time of follow-up was 19.5 ± 10.6 years (range 0.5–39). Histologically, bilateral tumors were present in 72.7% patients (*n*  = 24), thyroid capsular invasion was documented in 57.6% (*n*  = 19), 45.4% (*n*  = 15) had extrathyroidal extension, and 42.4% (*n*  = 14) had lymphovascular invasion. Most patients were staged pT3 (42.4%, *n*  = 14) and pN1 (81.8%, *n*  = 27). Median lymph nodes resected were 16. None of the patients showed distant metastases at presentation. All patients were treated at least once with ^131^I. During follow-up, four patients (14.8%), with persistent neck disease, were diagnosed with distant metastases, all of them in the lung. Two patients (1.8%) presented recurrent disease in the neck after being considered with no evidence of disease. At the last appointment, 18 patients (54.5%) were in remission, 4 (12.1%) had biochemical evidence of disease, 6 had structural disease, and for 5 patients disease status was considered as undetermined. There was no disease related mortality.

**Discussion/conclusion:**

Our study confirms that DSV-PTC is diagnosed more often in young patients and exhibits a local extensive disease at presentation. On the other hand, even in the presence of distant metastases, no patient died during follow-up.

## Introduction

Papillary thyroid carcinoma (PTC) is the most common thyroid cancer, accounting for more than 85% of the thyroid malignant lesions (1). An increase of its prevalence is recognized worldwide, mainly due to overdiagnosis of clinically irrelevant lesions, in part because of the greater use of more accurate imaging techniques (1). More than 15 histopathologic variants of PTC have been described, based on specific growth pattern, cell type, and stromal changes (2, 3). Although rare, tall cell, columnar cell, solid, hobnail, and diffuse sclerosing variants are usually associated with more aggressive clinical behavior (2). Diffuse sclerosing variant of PTC (DSV-PTC) is an uncommon subtype of thyroid cancer accounting for less than 6% of PTC (4). It is characterized by diffuse involvement of the gland, typically affecting both lobes, lymphovascular invasion, extensive squamous metaplasia, abundant psamomma bodies, marked stromal fibrosis, and prominent lymphocytic infiltration on histopathology evaluation (2, 3, 4). Debate regarding the prognosis of DSV-PTC is not closed. Several studies were published in the last years, comparing outcomes and prognosis of this variant to the outcomes of classic and high-risk variants of PTC. However, even more recent meta-analyses are not concordant regarding the prognostic significance of this rare variant (5, 6).

## Materials and methods

We retrospectively reviewed all the patients diagnosed with DSV-PTC admitted to the Endocrine Department of the Portuguese Institute of Oncology in Lisbon, between 1981 and 2020. All patients underwent neck surgery. Central compartment with or without lateral compartment lymph node dissections were performed when pre-surgical neck ultrasound (US), cytological evaluation, or surgical exploration identified lymph node disease. Histological specimens were analyzed according to World Health Organization (WHO) classification criteria. All patients were treated with ^131^iodine (I) after surgery (under recombinant thyroid-stimulating hormone (TSH) or L-thyroxine withdrawal). Periodic follow-up included, at least, TSH, thyroglobulin (Tg), and antithyroglobulin (anti-Tg) antibody determination, as well as neck US. Other imaging and/or functional tests were performed when relevant to the follow-up. Positive Tg was defined as any value above the sensitivity threshold of the assays used over the different years of the study.

Persistent or recurrent disease was defined as abnormal suppressed and/or stimulated Tg, raising anti-Tg antibodies, or evidence of loco-regional or distant metastases.

For each patient, data was recorded regarding age, gender, signs/symptoms at presentation, biochemical parameters, US and cytological findings, type, extension and number of surgical procedures, pathological data, post-operative diagnostic radioactive iodine (RAI) scanning, corresponding American Joint Committee on Cancer (AJCC) 8thedition pTNM staging system and American Thyroid Association (ATA) risk stratification (7), data from structural and biochemical follow-up, and other treatments required.

In the last follow-up appointment, patients were classified as no biochemical or structural evidence of disease (NED), biochemical evidence of disease (BED) (referring to abnormal Tg values in the absence of localizable disease or persistent anti-Tg antibodies), structural or functional evidence of loco-regional or distant metastases (SED), according to criteria presented by ATA in 2015 (8).

For statistical analysis, nonparametric continuous data were depicted as median values and normal distributed quantitative data as mean ± s.d. Categorical data were expressed as percentages. Statistical analysis was performed by means of SPSS program version 21.0®.

Our results were compared with those published in other studies focusing on DSV-PTC, obtained in a Medline search (1989–2020).

## Results

The clinicopathological features of DSV-PTC are summarized in Table 1. Thirty-three patients were evaluated. They were mainly women (78.8%) and mean age at presentation was 29.4 ± 11.7 years. The youngest patient was 10 years old and the oldest 65 years old, and only two patients were above age 45. Fourteen out of 33 had positive anti-Tg antibodies before surgery and six had positive anti-thyroid peroxidase, but only three patients (9.1%) were hypothyroid before surgery. In 28 patients (84.8%), cervical complaints (goiter/thyroid nodule or adenopathy) led to the diagnosis, and in the remaining patients, the diagnosis of thyroid nodules was incidental in routine medical examinations.

**Table 1:** Clinicopathological Features of DSV-PTV at diagnosis.

Variables	
Sex, *n* (%)	
Female	26 (78.8)
Male	7 (21.2)
Age (years), x ± s, min–máx	29.4 ± 11.7, 10–65
Positive TPO antibodies (*n* = 11), *n* (%)	6 (66.7)
Positive anti-Tg antibodies (*n* = 33), *n* (%)	14 (42.4)
Hypothyroidism (*n* = 33), *n* (%)	3 (9.1)
Palpable goiter/adenopathy (*n* = 29), *n* (%)	28 (84.8)
Goiter	20 (60.6)
Adenopathy	8 (24.2)
Previous cytological data suspicious/diagnostic of malignancy (*n* = 21), *n* (%)	21 (100)
Bilaterality (*n* = 33), *n* (%)	24 (72.7)
Diffuse pattern (uni/bi) (*n* = 33), *n* (%)	22 (71.4) (2/20)
Extrathyroidal extension (*n* = 29), *n* (%)	15 (51.7)
Lymphovascular invasion (*n* = 29), *n* (%)	14 (48.3)
Psammomatous bodies (*n* = 29), *n* (%)	9 (27.6)
Lymphocytic infiltrate (*n* = 29), *n* (%)	14 (48.3)
Thyroid capsular invasion (*n* = 29), *n* (%)	19 (65.5)
TNM staging (*n* = 33), *n* (%)	
T1a/1b	1 (3.0)/0 (0)
T2	7 (21.2)
T3	14 (42.4)
T4a/4b	4 (12.1)/1 (3.0)
Tx	6 (18.2)
N0	6 (18.2)
N1a/1b	7 (21.2)/20 (60.6)
Number of resected lymph nodes per patient, Med (Q__1__;Q_3_), min–máx (*n* = 26)	16.5 (1.5; 46), 0–74
Number of total metastatic lymph nodes per patient, Med (Q__1__;Q_3_), min–máx (*n* = 26)	9.5 (0; 16.8), 0–45

anti-Tg, antithyroglobulin; Bi, bilateral; Uni, unilateral.

Fine-needle aspiration biopsy (FNAB) of the thyroid gland or the cervical lymph nodes was performed in all patients but was available only in 21 (63.6%) patients. Three (14.3%) had the diagnosis of follicular tumor, nine (42.9%) of PTC (one of them DSV), and two (9.5%) suspicious for PTC and finally, six (28.6%) of them had the diagnosis of metastases of PTC and one (4.8%) was suspicious for PTC metastasis.

Total thyroidectomy was carried out in 25 (75.6%) patients and hemithyroidectomy in the remaining. In 25 (75.8%) patients, lymph nodes were also resected during this first surgery, in 23 (92.0%) from the central compartment and in 12 (48%) from the lateral compartments either unilateral in 7 (58.3%) or bilateral in 5 (41.7%). Lymph-node metastases in the upper mediastinum were resected in six (18.1%) patients. In three (9.1%) patients, focal ‘berry-picking’ lymphadenectomies were performed.

A second surgery was needed in 13 (39.4%) patients, in 3 to complete thyroidectomy and in the remaining because of persistent (7 patients) or recurrent disease (3 patients). A third surgery was performed in four (12.1%) patients, two with persistent and two with recurrent disease, and a fourth surgery in one patient (recurrence).

Thirteen patients (39.4%) suffered surgical complications, namely permanent hypoparathyroidism in 11 (33.3%), recurrent nerve palsy in 5 (15.2%), and spinal nerve palsy in 1 patient (3.0%). In two (6.1%) patients, recurrent nerve lesion was bilateral and tracheostomy was required. Two (6.1%) patients had two of the above complications and one (3.0%) patient had three.

**Figure 1:**
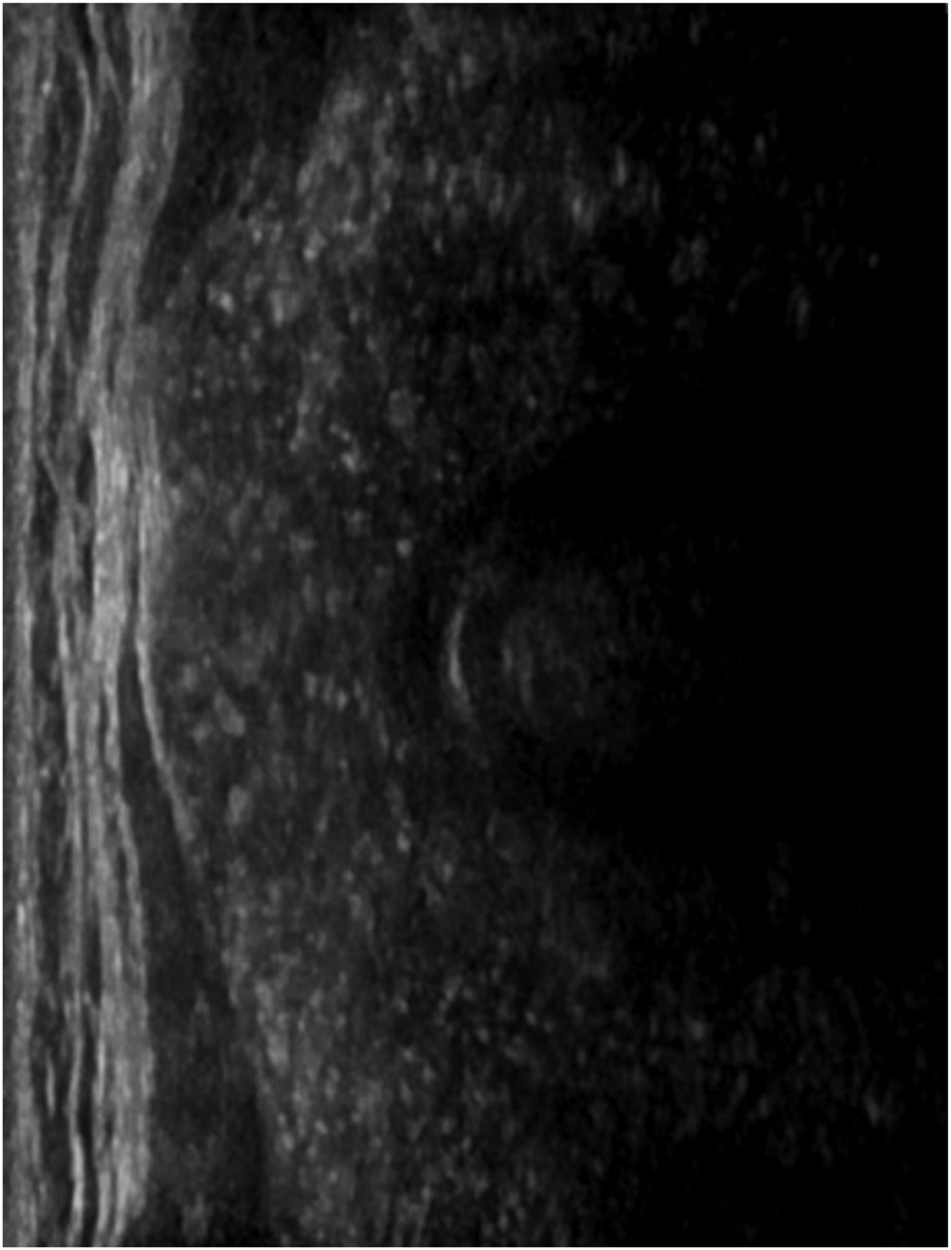
A 19-year-old male with diffuse sclerosing variant of papillary carcinoma of the thyroid gland with scattered microcalcifications throughout the thyroid gland.

**Figure 2:**
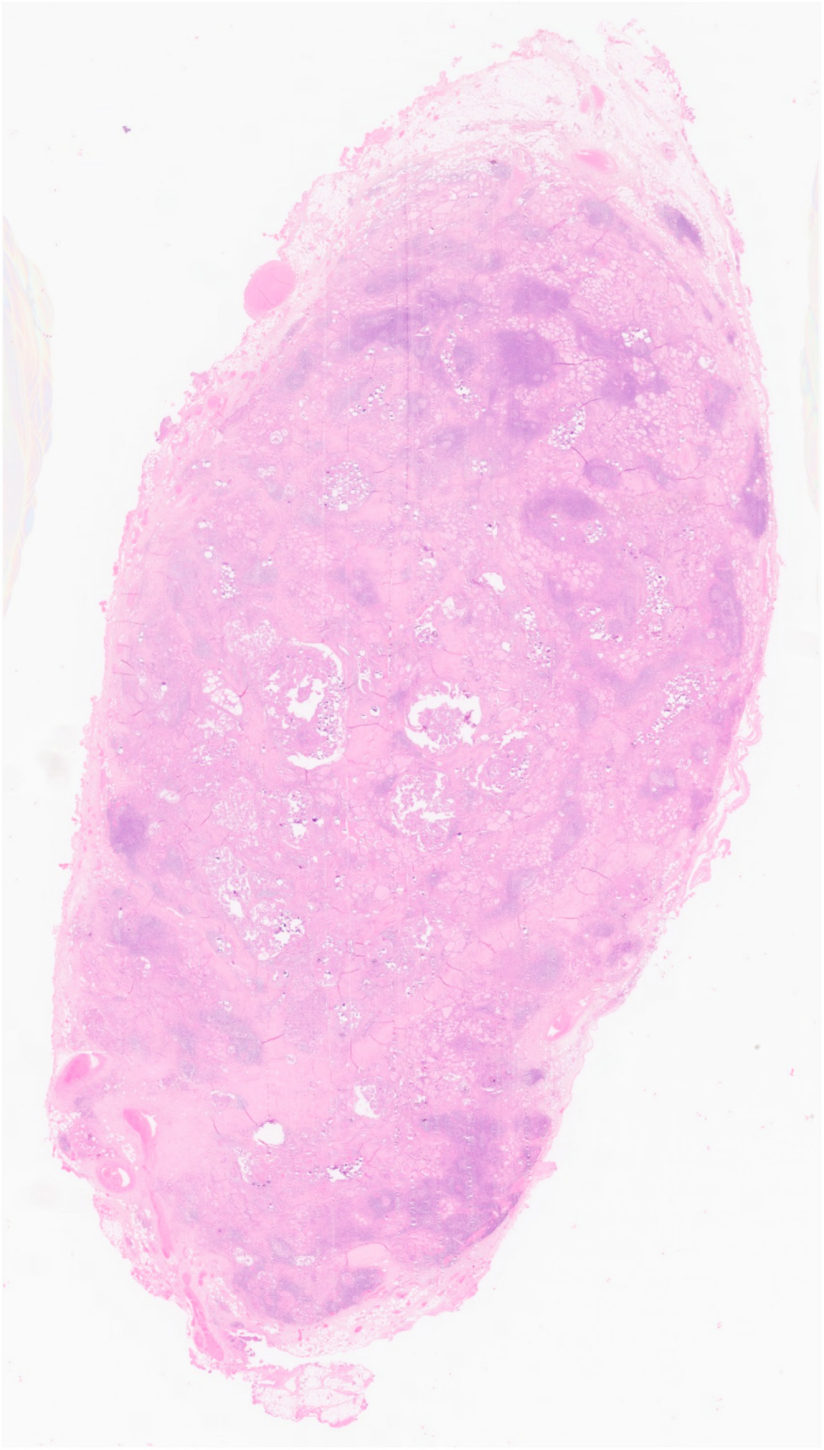
A diffuse pattern of infiltration, with fibrosis and lymphocytic infiltrate is apparent on low power view.

**Figure 3:**
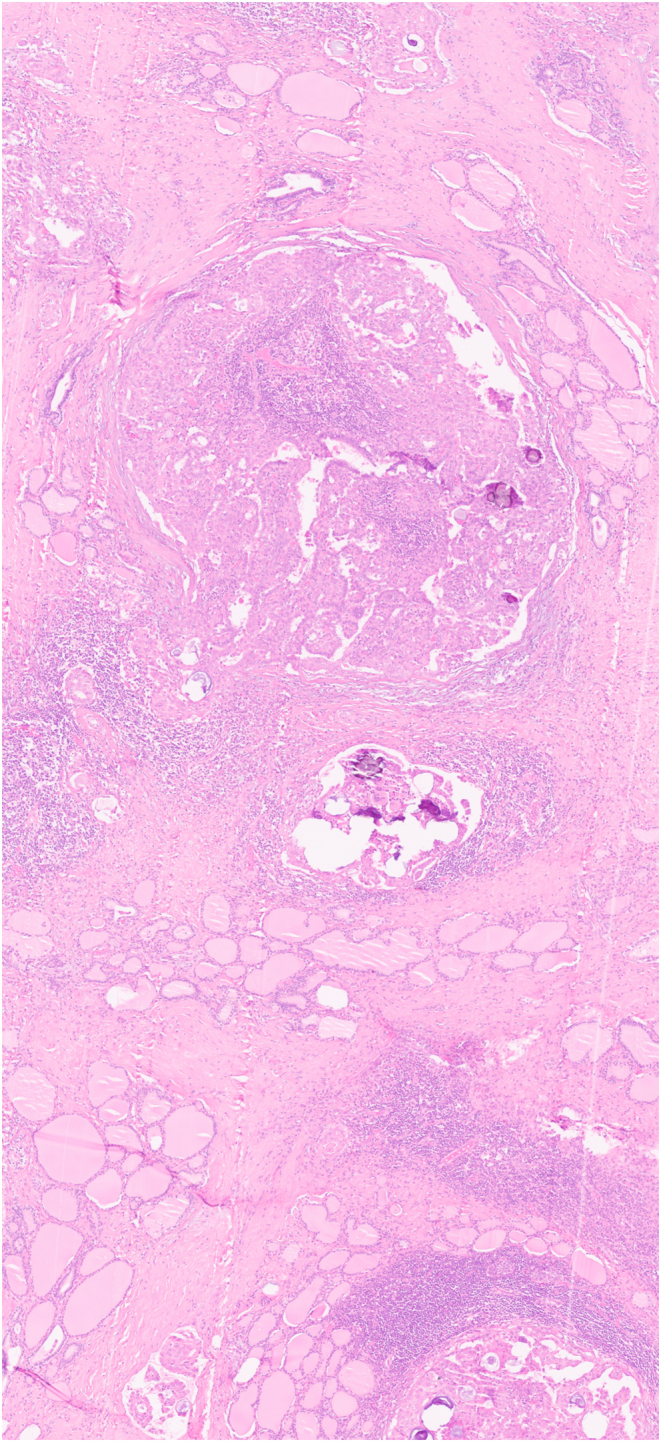
Magnififed view of an area of a diffuse sclerosing variant of papillary thyroid carcinoma: there is a diffuse infiltration of thyroid tissue with bands of fibrous tissue, lymphocytic infiltrate, numerous psammoma bodies and focal squamous metaplasia.

Pathological information was available in all 33 patients (Table 1). In 24 of them (72.7%), the tumor involved both thyroid lobes (shown in Fig. 1), either in a diffuse pattern (20 patients) or as nodular lesions (in 4 patients). In the remaining nine patients, the tumor involved only one thyroid lobe. When nodular lesions were identified, tumor diameters ranged between 0.5 and 6 cm. Capsular invasion was documented in 19 patients (57.6%), extrathyroidal extensionin 15 patients (45.5%), and lymphovascular invasion in 14 patients (42.4%). Regarding specific tissue characteristics of DSV-PTC, 8 patients (24.2%) presented psammomatous bodies and 14 (42.4%) lymphocytic infiltrates (shown in Figs 2 and 3).

Data obtained from pathological examination and classified according to the 8th edition of AJCC pTNM staging system is specified in Table 1. Most patients were pT3 (14, 42.4%) and pN1 (27, 81.8%). Median lymph nodes resected were 16 (min: 0; max: 74) and median positive lymph nodes per patient were 9. No patients presented distant metastases at presentation. The baseline ATA 2015 stratification risk is showed in Table 2, where 21 patients were classified as ATA intermediate risk of structural disease recurrence and 12 patients were classified as ATA high risk.

**Table 2:** Association between ATA risk groups and the revaluation between 12 and 24 months of follow-up.

ATA risk group/reavaluation betwwen 12 and 24 months	SED (*n* = 6)	BED (*n* = 3)	Indeterminate (*n* = 5)	NED (*n* = 17)
ATA intermediate risk (*n* = 21)	4 (19.0%)	4 (19.0%)	2 (9.5%)	11 (52.5%)
ATA high risk (*n* = 12)	4 (33.3%)	3 (25.0%)	2 (16.7%)	3 (25.0%)

ATA, American Thyroid Association; BED, biochemical evidence of disease; NED, no biochemical or structural evidence of disease; SED, structural or functional evidence of loco-regional or distant metastases.

All patients were treated at least once with ^131^I; median number of treatments was one (Q1;Q3: 1.0; 3.0). Sixty percent of patients received only one treatment. One patient received 11 treatments. Median total activity was 130 mCi (Q1;Q3, 100;395) (min: 40; max: 1195).

Regarding the first treatment with ^131^I, the mean administered activity was 99 ± 33 mCi (min: 40; max: 155), under recombinant TSH (16 patients) or L-thyroxine withdrawal (17 patients). Stimulated Tg was positive (>1 ng/mL) in 24 out of 31 patients (72.7%) and anti-Tg was positive in 16 out of 30 (48.5%), and in 9 patients, both markers were positive.

The mean follow-up was 19.5 ± 10.6 years (range 0.5–39). Four patients were diagnosed with distant metastatic disease during follow-up at 11, 17, 22, and 114 months after surgery, all of them in the lung, and detected by whole-body scan following RAI treatment in three patients. These four patients received total activities of ^131^I of 578, 1195, 804, and 470 mCi, respectively. They were followed for 96, 253, 417, and 404 months and, at the last follow-up appointment, two were considered as NED and the other two as SED due to lung metastasis but with stable Tg levels and stable tumor burden.

Two patients (6.1%) presented recurrent disease in the neck after being considered in NED. They were surgically treated and both patients were in NED at the last visit, 188 and 97 months after the first surgery. The association between ATA risk groups and the revaluation between 12 and 24 months of follow-up from all patients is presented in Table 2 (*P*  > 0.05). Only one patient was submited to cervical radiotherapy with 60 Gy due to persistent loco-regional disease and at the last follow-up appointment is considered as NED.

At the last appointment, 18 patients (54.5%) were classified as NED, 4 (12.1%) as BED, 6 as SED (4 loco-regional metastases and 2 distant metastases), and for 5 patients disease status was considered as undetermined. There was no disease related mortality. The association between ATA risk groups and the state of disease of disease at the final follow-up is shown in Table 3 (*P*  > 0.05).

**Table 3:** Association between ATA risk groups and the state of disease of disease at the final follow-up.

ATA risk group/state of disease at final follow-up	SED (*n* = 6)	BED (*n* = 3)	Indeterminate (*n* = 5)	NED (*n* = 17)
ATA intermediate risk (*n* = 21)	3 (14.3%)	1 (4.8%)	4 (19.0%)	13 (61.9%)
ATA high risk (*n* = 12)	3 (25.0%)	3 (25.0%)	1 (8.3%)	5 (41.7%)

ATA, American Thyroid Association; BED, biochemical evidence of disease; NED, no biochemical or structural evidence of disease; SED, structural or functional evidence of loco-regional or distant metastases.

## Discussion/conclusion

We analyzed a series of 33 patients diagnosed with DSV-PTC, treated and followed-up in one single Portuguese Institution for a mean period of 19.5 ± 10.6 years. As far as we know, this is the longest follow-up ever performed to patients with this PTC variant.

In agreement with previously reported studies, patients were mainly women (9, 10, 11, 12, 13, 14, 15, 16, 17, 18) and mean age was under 30s (range 10–65 years) (10, 11, 15, 16, 17, 18, 19, 20, 22, 23, 24, 25, 26, 27, 28, 29).

Because of the diffuse involvement of the thyroid gland that usually characterizes this variant, previous hypothyroidism was diagnosed in 9% of patients, similar to the prevalence of 6–10% reported in other studies (10, 11). Regarding the thyroid antibodies status at diagnosis, a significant number of patients were diagnosed with Hashimoto thyroiditis (42.4%). Hashimoto thyroiditis has also been reported in association with 30–75% DSV, but this feature was neither correlated with disease recurrence nor with mortality (2, 21, 24).

The majority of our patients reported neck complaints that initiated the diagnostic approach. Others found similar results (10, 11, 16, 18).

In our study, FNAB was suspicious or diagnostic of malignancy in 91% of cases. Other retrospective studies report similar findings (86–100%) (9, 10, 11, 16, 18). However, in our series, FNAB was diagnostic of DSV-PTC in only one patient. Specific diagnosis of DSV may prove challenging since lymphocytes infiltrate and the atypical follicular cells may be easily misdiagnosed as chronic thyroiditis (4). Besides, nuclear features of PTC are often less prevalent in DSV (4).

A high rate of surgical complications (39%) was observed, mainly permanent hypoparathyroidism (*n* = 11 (33.3%)), and this last complication is probably a consequence of the frequent lymph node invasion requiring extensive dissection of the central compartment.

A loco-regional aggressive behavior was found in our series with high prevalence of bilateral disease, thyroid capsular invasion, lymphovascular invasion, and lymph node metastases. These characteristics are also reported by the majority of the published series (9, 10, 11, 14, 23, 24, 25). During follow-up, in four patients (15%) with persistent disease, pulmonary metastases were detected. Lung is the most frequent metastatic location reported for DSV (9, 10, 12, 16, 17, 18, 19, 20) and a significant increase of risk is recognized compared to more common-PTC variants (9, 14, 23, 25). Only two patients presented recurrent disease after a period in NED, one of them in two different occasions. It is difficult to compare our results with other series since persistent and recurrent disease are usually presented together. None of the recurrences was a distant metastatic site.

In the last years two meta-analyses focused on the prognostic significance of DSV variant of PTC. In 2016, Malandrino *et al.* (5) compared 587 DSV with 64,611 conventional PTC patients, all collected from 10 published studies. In 2017, Voung *et al.* (6) analyzed 732 DSV-PTC that were compared with conventional PTC patients, collected from 16 previous published articles. These meta-analyses shared seven studies, and both concluded that DSV-PTC exhibits a more aggressive behavior than classic (C)-PTC, including higher rate of vascular invasion, ETE, lymph node metastases, and distant metastases, as well as higher likelihood of relapse. However, the first study found that the risk of cancer-related mortality was not different between DSV and C-PTC (OR = 1.34; 95% CI = 0.76–2.38). On the other hand, the second study reported a worse overall survival for patients diagnosed with DSV-PTC. Regarding cancer-related death, our results are in agreement with data reported by Malandrino *et al.* in 2016, since no patient died because of thyroid cancer after a long follow-up (5).

Finally, in 2020, Ho *et al*. (29) analyzed 5447 aggressive PTC variants from hospital-based and population-based US cancer registries, including 415 DSV. They observed long-term heterogeneous survival outcomes for different PTC variants. Patients were compared to 35,812 C-PTC. They found higher likelihood of DSV to present larger tumor size, higher mean metastatic lymph node number, ETE, and M1 disease. When propensity score analyses was performed to correlate PTC subtype with overall survival, no difference was found between C-PTC and DSV. Limberg* et al.*(28) analyzed C-PTC and aggressive variants of PTC patients with and without any invasive pathologic characteristics to determine whether these features impacted on prognosis, and DSV with invasive features, was an independent predictor of overall worse survival (HR 1.3 (95% CI 1.0–1.7)) compared to C-PTC (28).

Our study has some limitations such as its retrospective nature and that several parameters were not available for all patients.

Our study confirms that DSV-PTC is diagnosed more often in young patients, exhibits a local aggressive behavior at presentation, including a high prevalence of N1 disease. However, even when distant metastases are present, this variant shows an indolent course and no case of death occurred in our study after a long follow-up.

## Declaration of interest

The authors declare that there is no conflict of interest that could be perceived as prejudicing the impartiality of the research reported.

## Funding

This work did not receive any specific grant from any funding agency in the public, commercial, or not-for-profit sector.

## Statement of ethics

The authors declare that the procedures were followed according to the regulations established by the Clinical Research and Ethics Committee and to the Helsinki Declaration of the World Medical Association. The authors declare that the manuscript, complete or in parts, does not infringe any copyright and does not violate any privacy rights. The authors declare that no experiments were performed on humans or animals for this investigation. The authors state that subject has given his written informed consent to publish their case, including publication of images.

## Author contribution statement

The authors declare that all authors had a substantial contribution for this manuscript. The authors declare that all authors approve the final version of the manuscript.
